# Rho-kinase regulates tissue morphogenesis via non-muscle myosin and LIM-kinase during *Drosophila *development

**DOI:** 10.1186/1471-213X-6-38

**Published:** 2006-08-01

**Authors:** Valerie Verdier, Guang-Chao-Chen, Jeffrey Settleman

**Affiliations:** 1 Massachusetts General Hospital Cancer Center and Harvard Medical School, 149 13^th ^Street, Charlestown, MA 02129, USA

## Abstract

**Background:**

The Rho-kinases (ROCKs) are major effector targets of the activated Rho GTPase that have been implicated in many of the Rho-mediated effects on cell shape and movement via their ability to affect acto-myosin contractility. The role of ROCKs in cell shape change and motility suggests a potentially important role for Rho-ROCK signaling in tissue morphogenesis during development. Indeed, in *Drosophila*, a single ROCK ortholog, DRok, has been identified and has been found to be required for establishing planar cell polarity.

**Results:**

We have examined a potential role for DRok in additional aspects of tissue morphogenesis using an activated form of the protein in transgenic flies. Our findings demonstrate that DRok activity can influence multiple morphogenetic processes, including eye and wing development. Furthermore, genetic studies reveal that *Drok *interacts with multiple downstream effectors of the Rho GTPase signaling pathway, including non-muscle myosin heavy chain, adducin, and Diaphanous in those developmental processes. Finally, in overexpression studies, we determined that *Drok *and *Drosophila *Lim-kinase interact in the developing nervous system.

**Conclusion:**

These findings indicate widespread diverse roles for DRok in tissue morphogenesis during Drosophila development, in which multiple DRok substrates appear to be required.

## Background

Rho-kinases (also known as ROKs or ROCKs) were the first Rho GTPase-binding effectors to be identified and were initially characterized as mediators of the formation of RhoA-induced stress fibers and focal adhesions [1,2]. ROCKs are serine-threonine kinases that are most homologous to myotonic dystrophy kinase (DMPK) and citron kinase. They are comprised of a kinase domain at the N-terminus, followed by a coiled-coil domain containing a Rho-binding domain and a Pleckstrin-homology domain (PH) [3].

In non-muscle cells, ROCKs control a variety of cellular processes downstream of Rho, many of which depend upon actin cytoskeleton organization and cell contractility. These include cell-matrix and cell-cell adhesion, cell migration, neurite retraction and outgrowth, and cytokinesis.

Expression of a dominant-negative form of ROCK or treatment of cells with the selective pharmacologic ROCK inhibitor Y-27632 inhibits LPA-induced and Rho-induced formation of actin stress fibers and focal adhesions, implicating ROCKs in Rho-dependent signaling pathways to the cytoskeleton [3].

Several downstream substrates of ROCK that mediate such biological activity have been identified. The regulatory myosin-light-chain (MLC) of the protein myosin II is one substrate that is essential in regulating actomyosin contractility [4,5]. MBS, the myosin-binding subunit of the myosin-light-chain phosphatase (MLCP) has also been established as a mediator of ROCK function [6]. MLCP dephosphorylates MLC, implicating it as a negative regulator of acto-myosin contractility. ROCK phosphorylates MBS, consequently inhibiting its phosphatase activity and resulting in higher MLC phosphorylation [7]. Thus, there is a dual regulation of myosin II phosphorylation by ROCK; i.e., directly through MLC and through MBS, to exert its biological effects on actomyosin contractility.

Another ROCK substrate implicated in actin dynamics is LIMK (Lim-kinase). LIMKs are serine/threonine kinases that can regulate actin filament assembly. They are directly phosphorylated by ROCK, consequently increasing LIMK's kinase activity towards cofilin, an actin-binding and -depolymerizing protein in its unphosphorylated state, and which regulates the turnover of actin filaments [8,9].

In light of its prominent role in Rho-dependent cytoskeletal dynamics, ROCK function has also been studied in the context of tissue morphogenesis in several multicellular model organisms where it has been implicated in various developmental processes, including organogenesis in higher vertebrates such as chicken and mouse [10], embryo elongation and cytokinesis in *C. elegans *[11-13], and gastrulation in zebrafish [14]. ROCK has also been shown to function downstream of the Wnt/planar cell polarity pathway to ensure convergent extension cell movements during vertebrate gastrulation in the *Xenopus *embryo [15].

**Figure 1:**
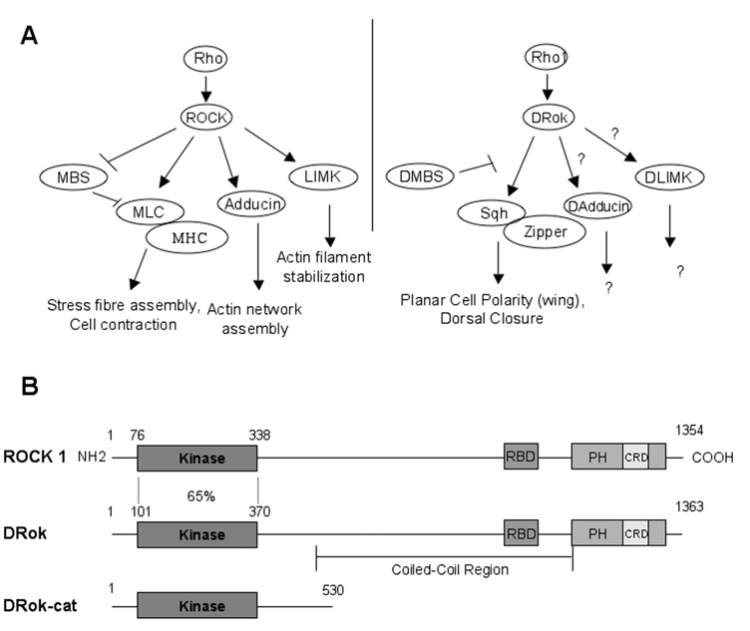
**Mammalian and *Drosophila *ROCK proteins**. (A) Schematic representation of several major ROCK substrates and the functions they mediate either in mammalian cells or in *Drosophila *tissues. (D)MBS: (*Drosophila*) Myosin Binding Subunit of Myosin Phosphatase; MLC: Myosin Light Chain; MHC: Myosin Heavy Chain; LIMK: LIM kinase; Sqh: *Drosophila *non-muscle Myosin Light Chain; Zipper: *Drosophila *non-muscle Myosin Heavy Chain. (B) The structure of mammalian and *Drosophila *ROCK proteins. The N-terminal kinase region of DRok, *Drok-cat *(amino acid 1 to 530) was isolated to express a contitutively active form of DRok. RBD: Rho-Binding Domain; PH: Plekstrin-Homology domain; CRD: Cysteine-Rich Domain.

In *Drosophila*, there is a single ROCK ortholog, DRok, which is highly conserved across all domains. DRok has been established as a downstream effector of *Drosophila *Rho1 [16]. DRok can phosphorylate Sqh, the *Drosophila *homolog of mammalian MLC, both *in vitro *and *in vivo *[16,17]. Unlike in mammalian cells, dual regulation of Sqh phosphorylation, by both DRok and *Drosophila *MBS (DMBS), has not yet been demonstrated yet, although DMBS has been shown to genetically antagonize the Rho1-DRok-Sqh signaling pathway during processes such as eye development and dorsal closure [18,19]. In addition, overexpression studies of full-length DRok in developing embryos have established a role for DRok in dorsal closure, a Rho1-mediated morphogenetic process [19]. Analysis of somatic clones of *Drok*^2^, a loss-of-function mutation of *Drok*, revealed a role for DRok in the highly conserved Frizzled-Dishevelled pathway that controls planar cell polarity. Thus, *Drok*^2 ^mutant clones exhibit tissue polarity defects resulting in an abnormal number of wing hairs and improper orientation of photoreceptor clusters in the eye [16]. In this developmental context, DRok's ability to regulate acto-myosin contractility through the control of MLC phosphorylation appears to account largely for its biological function. Figure 1A illustrates several major ROCK substrates, including the ones described above, and the cellular functions they mediate either in mammalian cells or in *Drosophila *development.

Here, we describe studies to address DRok-mediated signaling pathways in various aspects of tissue morphogenesis in developing *Drosophila*. By expressing a constitutively activated form of DRok, we observed disruption of normal development in various tissues, and these phenotypes can be suppressed by reducing the activity of known Rho pathway components through genetic interactions, suggesting that the observed phenotypes reflect functions of endogenous DRok. In an unbiased genetic screen to identify suppressors of a DRok-induced developmental defect, we isolated several alleles of a non-muscle myosin heavy chain ortholog (*zipper*). We also found that the Rho-kinase substrate, Lim-kinase, is likely to mediate functions of DRok in the developing nervous system. These studies collectively reveal that DRok mediates multiple aspects of tissue morphogenesis during development through multiple downstream effectors.

## Results and discussion

### Expression of a constitutively active form of DRok in various *Drosophila *tissues results in morphogenesis defects in developing tissues

The full-length ROCK protein is folded in such a way that the C-terminus binds to and negatively regulates the kinase activity of the N-terminus [20]. Therefore, to investigate downstream functions of DRok activity, we generated a mutant form of DRok predicted to be constitutively active, which consists of the isolated kinase region (DRok-cat for DRok-catalytic), sharing 65% identity with the corresponding isolated domain of mammalian ROCK1 (Fig. 1B). The latter has been previously reported to function as a constitutively active protein [21]. The activated form of DRok was expressed in various tissues of transgenic flies, under the control of a UAS promoter, using the UAS-GAL4 transactivation system, or directly in the eye under the control of the eye-specific GMR-promoter.

Numerous GAL4 drivers were used to express DRok-cat in the context of three different UAS-DRok-cat insertion sites (T5A, T1A, T2A) in various tissues throughout development. The results are summarized in Table 1. First, it is worth noting that many of the DRok-cat expression-induced phenotypes are dosage-sensitive; i.e., the phenotypes vary and increase in severity with increased expression of DRok-cat. Comparison among the the transgenics with varying DRok-cat expression levels was determined relative to the eye color marker intensity corresponding to each insertion. Expression of DRok-cat using the T2A insertion consistently leads to lethality with the exception of expression in the eye, using *eyeless*-Gal4, or in the wing margin with *Cy6*-GAL4. This suggests that excessive DRok activity leads to developmental defects in a variety of tissues. However, the T5A insertion seems to provide a sensitized genetic background suitable for analysis of some DRok-cat expression-induced visible effects in *Drosophila*, as expression of DRok-cat from that insertion, in various tissues, results in developmental phenotypes, but does not generally produce lethality.

**Table 1:** Phenotypes generated by different expression levels of DRok-cat in many *Drosophila *tissues

GAL4 driver	Expression pattern	T5A	T1A	T2A
*actin5c*-GAL4	ubiquitous	lethal	lethal	lethal
*tubulin*-GAL4	ubiquitous	lethal	lethal	lethal
*da*-GAL4	Early embryo	lethal	lethal	lethal
*prd*-GAL4	Early embryo	lethal	lethal	lethal
*69B*-GAL4	3^rd ^instar larval discs	Viable. Rough eyes	Viable. Rough eyes	lethal
LE-GAL4	Leading-edge epidermis	Viable no phenotype	Viable no phenotype	lethal
*elav*-GAL4	CNS	Viable no phenotype	Viable no phenotype	Semi-lethal
*1407*-GAL4	CNS/PNS	Viable no phenotype	Viable no phenotype	lethal
*en*-GAL4	Wing discs-posterior half	Missing crossveins	lethal	lethal
*Cy6*-GAL4	Wing discs-margin	Viable no phenotype	Viable no phenotype	Notched wings
*eyeless*-GAL4	Eye discs	Slightly rough eyes	Slightly rough eyes	Rough eyes
*GMR*-GAL4	Eye discs	Rough eyes	Rough eyes	Rough eyes

T5A, T1A and T2A correspond to independent genomic insertions of the *UAS-Drok-cat *transgene with distinct levels of expression (T5A: low expression, T1A: intermediate expression, T2A: high expression)

In addition, it is worth mentioning that although it is formally possible that phenotypes generated by expression of *GMR-Drok-cat *could result from non-specific secondary effects due to the engineered expression of the mutant DRok-cat protein, the biological relevance of these phenotypes has been demonstrated and will be described below with the analysis of genetic interactions between *Drok *and other components of the Rho GTPase pathway in this DRok-cat-expression model.

Ubiquitous expression of DRok-cat using *actin*-GAL4 or *tubulin*-GAL4 drivers results in lethality at embryonic or early larval stages, independently of the level of DRok-cat expression. Similar results are observed when DRok-cat is specifically expressed in early embryogenesis: *daughterless*-GAL4 (*da*-GAL4) and *prd*-GAL4 are two embryonic drivers that produce a larval or embryonic lethal phenotype when driving expression of DRok-cat. Targeted expression of DRok-cat to some tissues, such as third instar larval discs (*69B*-GAL4), epidermal leading edge cells in embryogenesis (LE-GAL4), or the central nervous system (*elav*-GAL4, *1407*-GAL4), results in lethality depending on the dosage of expressed DRok-cat. Together, these findings suggest that DRok activity must be tightly regulated during embryogenesis. Notably, genetic disruption of *Drok *(a null allele of the gene) has previously been reported, and zygotic mutant animals die at the larval stage, indicating that *Drok *is an essential *Drosophila *gene [16], and suggesting that excessive or insufficient DRok activity is incompatible with normal development.

### Excessive DRok activity disrupts both ommatidial structure and crossvein formation in the developing eye and wing, respectively

**Figure 2:**
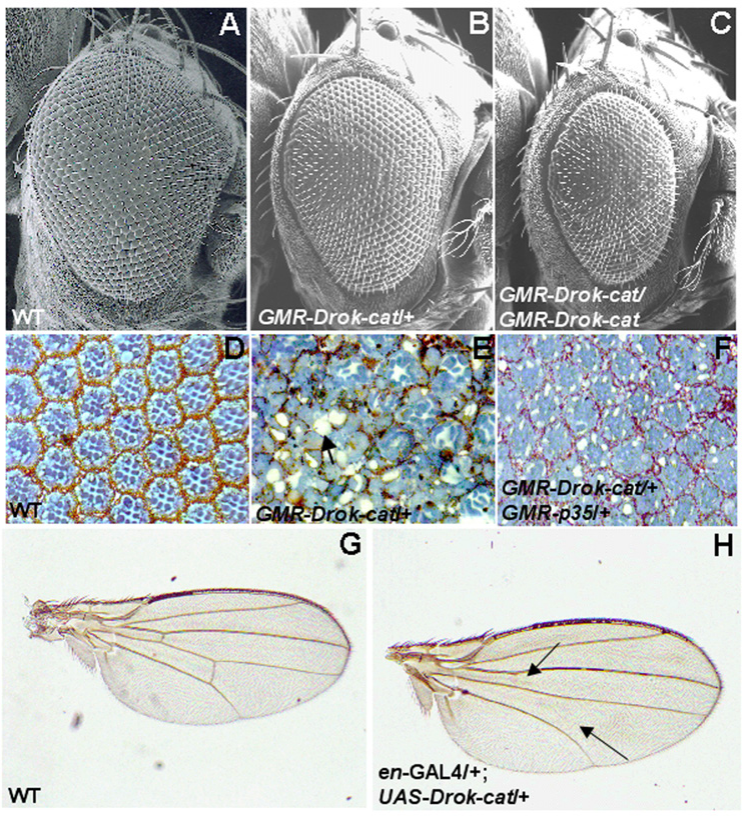
**Expression of activated DRok induces eye and wing defects**. (A-C) Scanning Electron Microscopy photographs of wild-type (A), one-copy *GMR-Drok-cat *transgenic (B) or two-copy *GMR-Drok-cat *transgenic (C) eyes. Expression of one copy of DRok-cat results in a slightly rough eye (B), of two copies in a rougher eye phenotype (C), which provides a basis for a convenient assay to identify genetic modifiers. (D-F) Tangential retinal sections of wild-type (D), one-copy *GMR-Drok-cat *transgenic (E) or one-copy *GMR-Drok-cat *and one-copy *GMR-p35 *(F) eyes. In the one copy-transgenic eye, the underlying retina is severely disrupted, associated with loss of cells (E, arrow). This phenotype can be rescued by overexpressing the baculoviral caspase inhibitor p35 (F). (G, H) Light microscopy photographs of a wild-type (G) or a *en-GAL4<UAS-Drok-cat *expressing (H) wing. Expression of DRok-cat in the posterior part of the wing results in the disappearance of the crossveins (H, arrows).

In the developing eye, expression of DRok-cat from the GMR promoter, which is induced upon binding of the transcription factor Glass during the 3^rd ^instar larval stage, results in eyes that exhibit a slight roughness and reduction in overall size compared to wild-types eyes (Fig. 2A, 2B). However, tangential retinal tissue sections of wild-type (Fig. 2D) and single-copy-*GMR-Drok-cat *(Fig. 2E) reveal that expression of DRok-cat results in a dramatic disruption of the ommatidial structure associated with apparent loss of cells resulting from cell death (Fig. 2E, arrow). This is further supported by the fact that GMR-mediated overexpression of the baculoviral caspase inhibitor p35 in the developing eye efficiently suppresses the mutant retinal phenotype induced by excessive DRok activity (Fig. 2F). Two-copy *GMR-Drok-cat *transgenic flies exhibit a more severe eye roughness associated with a significantly reduced size of the eye (Fig. 2C). We observed the same severe retinal phenotype with expression of *GMR-Drok-cat *from multiple transgene insertion sites, and it was not diminished or suppressed by specific GMR transcriptional suppressors (data not shown), indicating specificity of the strong phenotype to DRok-cat expression, independently of the insertion site. It seems that expression of DRok-cat does not result in planar polarity defects, even when cell death is prevented with co-expression of p35, as opposed to depletion of DRok in eye clones [16]. It is possible that expression of a constitutively active form of DRok, which probably functions in complex signaling networks, influences multiple downstream signaling pathways. Consequently, the overall resulting phenotype might not reveal planar polarity defects induced by one pathway. Another potential explanation for the absence of clear polarity defects in *GMR-DRok-cat, GMR-p35 *dissected samples is that only a small percentage of flies may exhibit these defects, in which case further investigation, including the quantification of abnormal eye polarity could be insightful. However, the strong external phenotype of *GMR-DRok-cat *flies is reminiscent of the previously reported DRho1 overexpression-induced eye phenotype [22], suggesting that the observed eye disruption reflects a specific dysfunction in regulation of a DRho1-DRok signaling pathway in the developing retina.

Expression of DRok-cat in the posterior half of the wing, using *en*-GAL4 as a driver, results in disappearance of the crossveins, suggesting that DRok may play a role in crossvein formation (Fig. 2H, arrows). Unlike the eye phenotype, this is not rescued by overexpression of p35 in the wing, indicating that the absence of crossveins is probably not due to apoptosis of crossvein cells (data not shown). In addition, expression of DRok-cat does not produce a wing hair polarity phenotype, unlike *Drok*^2 ^mutant clones [16]. As described above, it is possible that, upon expression of activated DRok in the posterior half of the wing, multiple integrated signaling pathways are activated to produce a visible crossvein phenotype rather than hair polarity defects. The presence or absence of crossveins in wing development has been reported to depend upon an inductive signal from the dorsal wing epithelium that is required for the formation of vein tissue in the ventral wing epithelium. That process has been shown to involve the products of the *crossvein *(*cv*), *cv-2*, *decapentaplegic *(*dpp*), *glass bottom boat *genes, and other components of the Bone Morphogenetic Protein (BMP)-like signaling pathway [23]. Therefore, to further characterize the DRok-cat crossveinless wing phenotype, we tested genetic interactions between *Drok *and the above genes but did not detect any interaction in the *en*-GAL4-induced DRok-cat expression background. This might reflect the fact that modifications of the DRok-cat phenotype, which is due to expression of a constitutively activated form, would only be expected using mutations that affect pathway components downstream of DRok.

### *Drok *genetically interacts with members of the Rho GTPase signaling pathways in the eye and in the wing

**Figure 3:**
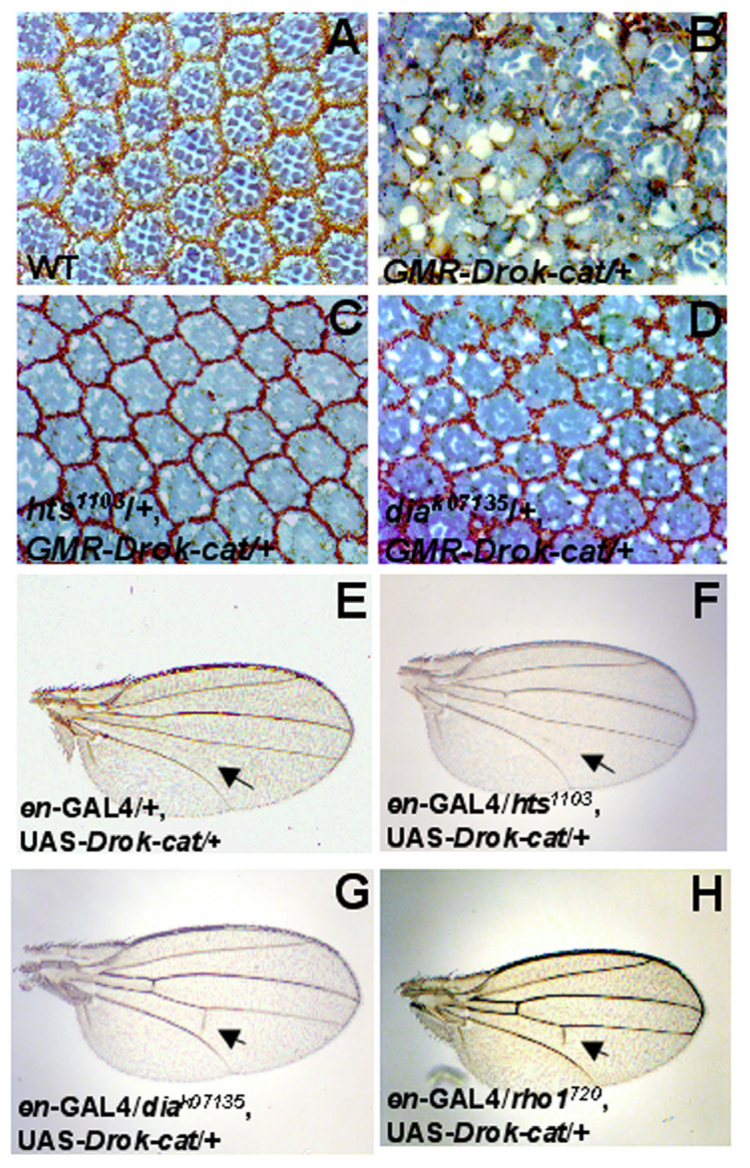
**Genetic interactions between *Drok *and several genes involved in Rho signaling pathways**. (A-D) Tangential retinal sections of eyes of the following genotypes: wild-type (A), one-copy *GMR-Drok-cat *transgenic (B), or one-copy *GMR-Drok-cat *and one mutant loss-of-function allele of either *adducin *(*hts1103*) or *diaphanous *(*dia*) (C or D, respectively). Taking away one copy of either *adducin *or *diaphanous *reverts the *GMR-Drok-cat*-induced eye phenotype to a near to wild-type appearance. (E-H) Light microscopy photographs of a *en-GAL4<UAS-Drok-cat *expressing wing on its own (E) or *en-GAL4<UAS-Drok-cat *expressing wings in various heterozygous loss-of-function mutant backgrounds including *hts1103 *or *dia *or *rho1*^720 ^(F-H). Taking away one copy of *adducin *(*hts1103*) does not rescue the missing crossvein phenotype, whereas heterozygosity for one copy of *diaphanous *(*dia*), or for one copy of *rho1 *(*rho1*^720^) almost entirely rescues or partially rescues the wing phenotype, respectively.

To establish the biological relevance of DRok-cat-associated phenotypes observed in the eye and in the wing, we tested genetic interactions between *Drok-cat *and loss-of-function alleles of *Drosophila *orthologs of mammalian genes that are known to interact with mammalian Rho-kinase. In mammalian cells, Rho-kinase has been found to phosphorylate adducin (*Drosophila *Hts), thereby leading to its recruitment to the cortical meshwork of the cell [24]. Eliminating one copy of *hts *efficiently suppresses the GMR-DRok-cat-induced phenotype (Fig. 3C) but does not affect the DRok-cat expression-induced wing phenotype (Fig. 3F, arrow). An explanation for this might be that Hts is not expressed in the wing or that it simply is not involved downstream of DRok in the regulation of crossvein formation. On the other hand, suppression is detected in both the eye and the wing when disrupting one copy of *dia*, which encodes the *Drosophila *ortholog of mammalian Diaphanous, a Rho specific target protein involved in stress fiber formation [25] (Fig. 3D, 3G, arrow). There have been a few previous interesting reports about the somewhat complex relationship between ROCK and mDia. Whereas LIMK, a DRok substrate, and mDia have been shown to cooperate in the regulation of serum response factor and actin dynamics in PC12 cells [26], ROCK and Dia exhibit opposing effects on adherens junctions downstream of Rho in epithelial cells [27]. In our system, the genetic interaction between *Drok *and *dia *suggests that the gene products, DRok and Dia functionally cooperate in the fly eye or wing development. In addition, a loss-of-function mutation of *rho1*, *rho1*^720^, is able to partially suppress the wing phenotype, suggesting that DRok-induced biological effects in crossvein formation are partly mediated by a Rho1-dependent DRok-independent parallel pathway (Fig. 3H, arrow). However, deleting one copy of *rho1 *has no effect on the DRok-cat-induced eye phenotype (data not shown). Not only does this genetic analysis demonstrate the significance of DRok-cat-induced phenotypes in the eye and in the wing by verifying some predicted genetic interactions, but it stresses the fact that signaling pathways triggered by the expression of activated DRok are sensitized to gene dosage modification. As described below, this has enabled us to use the DRok-cat-induced phenotypes to screen for downstream genetic interactors of *Drok*.

### DRok interacts with zipper, the *Drosophila *non-muscle myosin heavy chain, in the eye and in the wing

**Figure 4:**
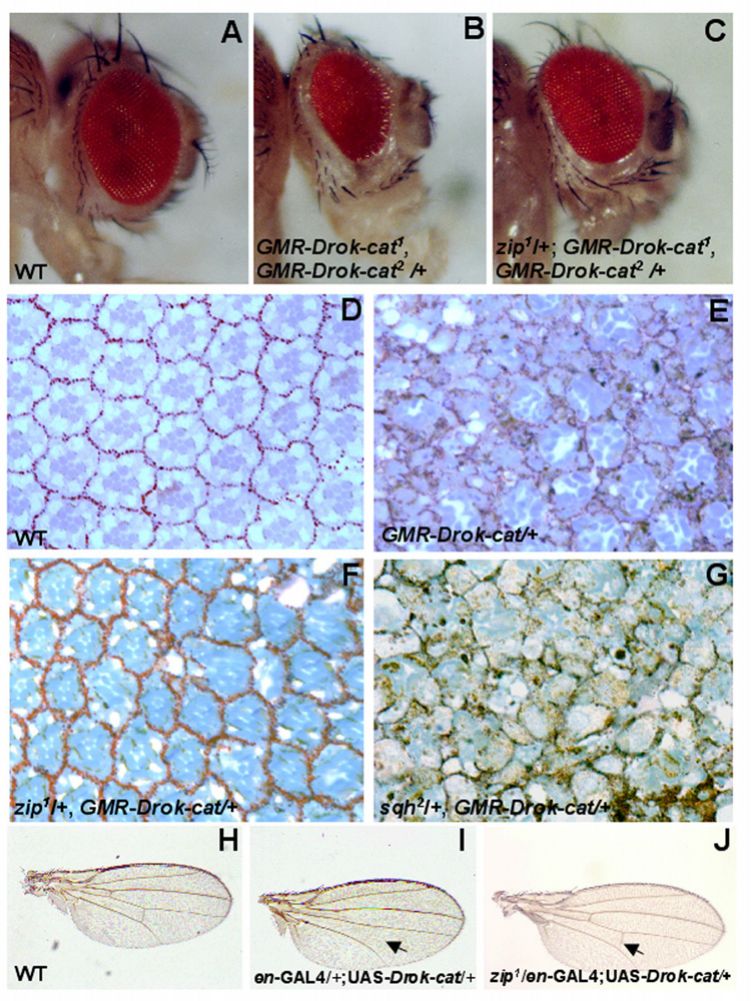
***zipper *as a genetic interactor of *Drok *in a screen for dominant suppressors of the DRok-cat expression-induced rough eye phenotype**. (A-C) Expression of two copies of the *GMR-Drok-cat *transgene (*GMR-Drok-cat*^1^, *GMR-Drok-cat*^2^) induces a rough eye phenotype associated with a smaller eye size (B) compared to a wild-type eye (A). This phenotype was dominantly suppressed by four independent EMS-induced mutations which all map to the *zipper *locus, the *Drosophila *non-muscle myosin heavy chain gene. The loss-of-function *zip*^1 ^mutation also rescued the *GMR-Drok-cat*^1^, *GMR-Drok-cat*^2^-induced eye phenotype (C). (D-G) Tangential retinal sections of eyes of the following genotype: wild-type (D), one-copy *GMR-Drok-cat *transgenic (E), or one-copy *GMR-Drok-cat *and one mutant loss-of-function allele of either *zipper *(*zip*^1^) or *spaghetti squash *(*sqh*^2^) (F or G, respectively). Unlike *zip*^1^, *sqh*^2^, a loss-of-function mutant of the *Drosophila *non-muscle myosin light chain, does not suppress the *GMR-Drok-cat*-induced eye phenotype. (H-J) Light microscopy photographs of a wild-type wing (H), a wing expressing *en-GAL4<UAS-Drok-cat *with missing crossveins (I, arrow) or a wing expressing *en-GAL4<UAS-Drok-cat *in a heterozygous *zip*^1 ^background (J). Heterozygosity for *zipper *did partially rescue the missing crossvein phenotype (J, arrow).

To identify novel components of a DRok-mediated signaling pathway, we performed a dominant modifier screen using ethyl-methane-sulfonate (EMS) as a mutagen. Flies carrying two copies of the *GMR-Drok-cat *transgene, which are associated with a consistent visible rough eye phenotype whose modification is easily detectable under light microscopy, were used as a starting point to identify mutants that can modify the phenotype (Fig. 4B). Because we were expressing a constitutively activated form of DRok in the eye, we expected to isolate mutations in downstream components of a DRok-mediated pathway or genes that encode proteins that function in distinct pathways but in cooperation with DRok. In a screen of ~12,000 mutagenized flies, we isolated four EMS-induced mutations that are each able to reproducibly and specifically suppress the DRok-cat expression-induced rough eye phenotype. We determined that these mutations are recessive lethal and correspond to a single complementation group based on lethality, suggesting that they likely represent mutations of the same gene or of different genes that are co-synthetic lethal, possibly functioning in the same signaling pathway. In order to identify the molecular nature of these suppressors, we undertook meiotic mapping and found that each mutation maps to the same genetic locus, i.e. the cytogenetic region 60 on the second chromosome. Moreover, a loss-of-function allele of *zipper*, *zip*^1^, the *Drosophila *non-muscle myosin heavy chain gene, which maps to this region, fails to complement each of the four suppressor alleles. In addition, heterozygosity for *zip*^1 ^suppresses the two-copy rough eye phenotype (Fig. 4C) and, seen with each of the suppressor mutations, is able to rescue the single copy-*GMR-DRok-cat*-induced ommatidial disruption (Fig. 4F). These mutations also partially rescue the DRok-cat-induced reduction in crossveins in the wing (Fig. 4J, arrow). Finally, phenotypic analysis of animals trans-heterozygous for each suppressor mutation reveals an embryonic lethality associated with a "dorsal open" phenotype similar to that of homozygous *zip*^1 ^mutant embryos [28] (data not shown). Taken together, these data strongly suggest that the isolated complementation group is comprised of loss-of-function alleles of *zipper*. Thus, in a screen for dominant suppressors of an activated DRok expression-induced rough eye phenotype, we have identified four new alleles of *zipper*, the *Drosophila *ortholog of the mammalian myosin heavy chain gene.

### *sqh*, unlike *zipper*, does not interact with constitutively activated *Drok*

As previously mentioned, Zipper is orthologous to members of the family of non-muscle myosin heavy chain proteins. Myosin heavy chains are chemo-mechanical motors which drive contraction of the actin cytoskeleton. These proteins bind to non-muscle myosin light chains (MLC) and their assembly forms myosin II molecules [29]. Rho-kinase has been reported to regulate the phosphorylation of the non-muscle myosin regulatory light chain (MLC), primarily at Ser-19 and secondarily at Thr-18 both *in vitro *and *in vivo*. Phosphorylation of MLC at these sites induces a conformational change that allows myosin II to form filaments and increases its actin-activated ATPase activity [3,4]. In *Drosophila*, the regulation by DRok of the phophorylation of Sqh, the *Drosophila *ortholog of MLC has also been demonstrated and Sqh and Zipper have been shown to both participate in establishing wing hair number in planar epithelial polarity [16]. In addition, *Drok*, *sqh *and *zipper *have been reported to interact genetically during another *Drosophila *developmental process, namely dorsal closure [19]. We therefore tested whether *sqh *also interacts with *Drok *in our overexpression system. Loss of one copy of the *sqh *gene (using the loss-of-function *sqh*^2 ^allele) does not suppress the two-copy *GMR-Drok-cat*-induced rough eye phenotype, and has no effect on the single-copy *GMR-Drok-cat*-induced ommatidial disruption (Fig. 4G). In addition, heterozygosity for *sqh*^2 ^does not even partially rescue the wing crossvein phenotype (data not shown). One possible explanation is that the inability of *sqh*^2 ^to rescue the wing phenotype or the *GMR-Drok-cat *eye phenotype is due to perdurance of maternal Sqh, which lasts longer than that of maternal Zipper in development: homozygous *sqh*^2 ^(null allele) mutant animals die at the 3^rd ^instar larval stage, whereas homozygous *zip*^1 ^(null allele) animals die at the embryonic stage. As a result, the absence of rescue of the wing or the *GMR-Drok-cat *eye phenotype by *sqh*^2^, not by *zip*^1^, may reflect a differential perdurance of Sqh and Zipper maternal products. Another possibility is that *Drok *might interact genetically with *zipper *independently of the *Drok-sqh *known interaction in a particular biological context. Thus, it is possible that DRok directly regulates Zipper in addition of phosphorylating Sqh. Unpublished studies from *Acanthamoeba castellanii *have shown that incorporation of ^32^Pi into crude extracts of *A. castellanii *myosin II heavy chain is significantly decreased in the presence of the Rho-kinase inhibitor Y-27632 or antibodies against ROCK, suggesting that Rho-kinase might be regulating the phosphorylation of myosin II heavy chain *in vitro*, in *A. castellanii*. However, in trying to address the biochemical nature of the genetic interaction between *Drok *and *zipper *in mammalian cells, we were unable to detect phosphorylation of nmMHC by Rho-kinase *in vitro *in a kinase assay or *in vivo *in a ^32^Pi incorporation assay, although several putative consensus sites for Rho-kinase phosphorylation have been found in the nmMHC amino acid sequence (data not shown). Thus, in addition to the *Drok-sqh-zip *interaction reported so far, there may be an interaction between Zipper and DRok via a Sqh-independent pathway, but this interaction may not be regulated by phosphorylation, rather by other signaling events. Interestingly, in the eye disc, DRok has been suggested previously as not being the major kinase responsible for the phosphorylation of Sqh, as phospho-Sqh staining was not altered in *Drok*^2 ^mutant clones [30]. Another kinase might phosphorylate Sqh specifically in the eye disc, which could explain the lack of detected genetic interaction between *Drok *and *sqh *in our system.

Overall, we have shown genetic evidence that levels of Zipper, the *Drosophila *non-muscle myosin heavy chain, limit the actions of DRok during *Drosophila *development, and that Zipper, in addition to Sqh, is an important downstream player in mediating DRok's biological effects during various developmental processes, such as in the eye and in the wing.

### DRok interacts with Lim-kinase in the developing nervous system

**Figure 5:**
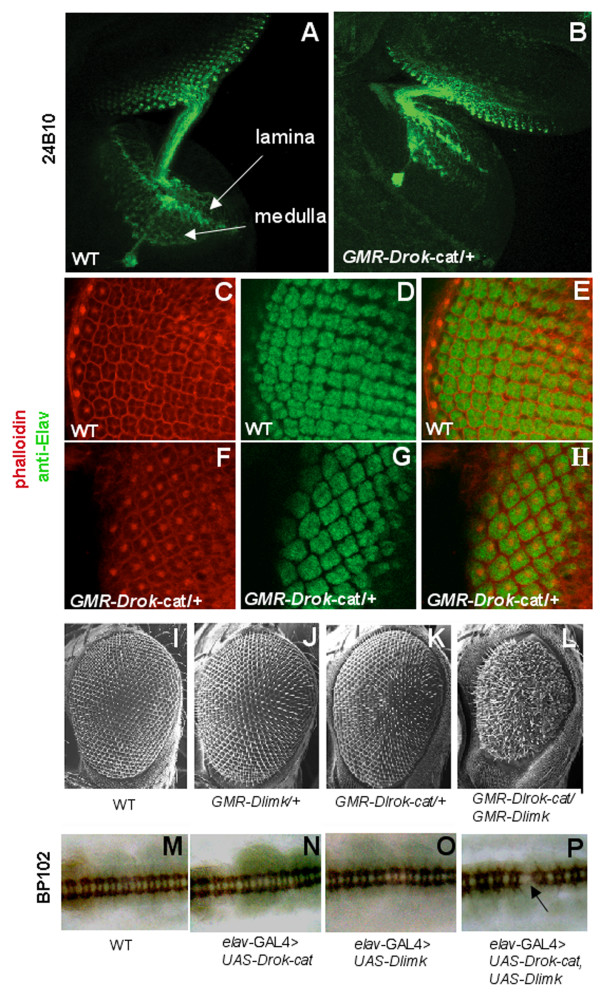
**DRok in axonal development**. (A, B) Immunostaining of either wild-type (A) or DRok-cat-expressing (B) photoreceptor neurons which send axonal projections from the developing 3^rd ^instar larval eye disc into the optic lobe of the brain. Axonal guidance and targeting appear normal in DRok-cat-expressing larval eye discs. *GMR-Drok-cat*/+ photoreceptors project correctly to the lamina and medulla layers into the optic lobe. In B, the axons are folded due to tissue mounting. (C-H) Double immunostaining of either wild-type (C-E), or *GMR-Drok-cat*/+ (F-H) 3^rd ^instar larval eye discs, with phalloidin (C, F) and an anti-Elav antibody (D, G) to detect actin and differentiating neurons, respectively. The overall morphology and differentiation pattern in photoreceptors is undistinguishable between wild-type and DRok-cat-expressing 3^rd ^instar larval eye discs. (I-L) Scanning electron microscopy pictures of wild-type (I), *GMR-Dlimk*/+ (J), *GMR-Drok-cat*/+ (K) or *GMR-Dlimk/GMR-Drok-cat *(L) eyes. Whereas overexpression of DLimk or expression of DRok-cat, separately, does not perturb the external morphology of the eye, co-expression of DLimk and DRok-cat results in a strong rough eye phenotype associated with decreased eye size. (M-P) Immunostaining of the embryonic CNS (BP102 antibody) of the following genotypes: wild-type (M), *elav-Gal4>UAS-Drok-cat *(N), *elav-Gal4>UAS-Dlimk *(O) or *elav-Gal4>UAS-Drok-cat, UAS-Dlimk *(P). As observed for the eyes, whereas overexpression of DLimk or expression of DRok-cat alone does not alter the proper organization of the embryonic CNS marked by adjacent patterns of connected neurons (M), co-expression of DLimk and DRok-cat leads to the disruption of connecting neurons (P, arrow).

The single copy-transgenic *GMR-Drok-cat *retinal phenotype led us to explore the role of DRok in the developing nervous system by analyzing the mutant photoreceptors earlier in development; i.e., in larval eye imaginal discs. Photoreceptor neurons differentiate from the developing eye disc and send axonal projections into the optic lobe of the brain via the optic stalk, more precisely, into a single superficial layer termed the lamina and a deep layer called the medulla [31] (Fig. 5A). The observation that DRok-cat-expressing differentiated photoreceptors in the adult eye are disrupted and collapsed raised the question as to whether their axons properly form and correctly project to the optic lobe earlier in development. Staining of single-copy *GMR-Drok-cat*-expressing 3^rd ^instar larval eye discs and associated optic lobes with 24B10, an antibody that specifically labels photoreceptor axons, reveals no major difference in axonal projection and targeting between wild-type and DRok-cat-expressing eye discs, indicating that proper axonal guidance and projection of photoreceptors are not affected by excessive DRok activity (Fig. 5B). Similarly, in a double anti-Elav and phalloidin staining of wild-type versus single copy *GMR-Drok-cat*-expressing 3^rd ^instar larval eye discs, the detection of newly differentiating neurons reveals no difference in the overall morphology and differentiation pattern between those two genotypes (Fig. 5C–H). Taken together, these results indicate that excessive DRok activity does not prevent proper development of 3^rd ^instar larval photoreceptors, and that DRok-cat expression-induced retinal defects must arise during later developmental stages. Consistent with this, when sectioning retinas from *GMR-Drok-cat*-expressing young (virgin) adult flies, we observed retinal disruption, although less substantial than that seen in *GMR-Drok-cat*-expressing older adult flies (data not shown), suggesting that excessive DRok activity is detrimental to the eye around the time of eclosion, or at the pupal stage. Thus, photoreceptor differentiation and development do not appear to require strict regulation of DRok activity, as DRok-cat-related retinal defects appear later in development, after differentiation of these neurons. However, DRok activity becomes critical for the maintenance of photoreceptor integrity. Moreover, it has been established from examination of *Drok*^2 ^somatic clones in the eye that DRok is required for the proper arrangement of photoreceptors and orientation of the ommatidia [16]. Altogether, our results and published data indicate a requirement for DRok and the regulation of its activity in development and maintenance of the fly eye. Secondly, since there is a time-dependent progression of the severity of the retinal neuronal phenotype, the observed DRok-induced photoreceptor disruption might reflect neuronal degeneration leading to neuronal death, a possibility that is further supported by the observed suppression of the DRok-cat-related retinal phenotype by overexpression of the baculoviral caspase inhibitor p35.

Among the Rho-kinase substrates that have been strongly implicated in neural development are the Lim-kinases. The single *Drosophila *Lim-kinase (DLimk) is required for proper synapse formation and proper regulation of its activity is necessary for normal axon growth [32,33]. To determine whether DRok-mediated activation of DLimk plays a role in proper neural development, we crossed transgenic flies expressing activated DRok with flies over-expressing DLimk to examine phenotypes in the developing nervous system. First, using GMR-driven transgenes to identify a potential interaction in the developing eye, we observed that while overexpression of DLimk causes no detectable effects on eye development (Fig. 5J), co-expression of DLimk with activated DRok results in a dramatic disruption of eye development associated with a severe morphology defect of the external eye and a reduced overall eye size (Fig. 5L). Since the effects of a single-copy DRok-cat transgene on exterior eye structures in this setting are relatively mild (Fig. 5K), this finding is consistent with a synergistic interaction between these two proteins, and suggests that a DRok-DLimk signal may be influencing normal development. Second, a similar synergistic interaction between DRok and DLimk was observed in the developing central nervous system. Using an *elav*-GAL4 driver to express UAS-linked *Drok-cat *and *Dlimk *in developing neurons, it was observed that neither protein alone causes any detectable effect on the appearance of the embryonic nervous system (Fig. 5N, 5O), whereas co-expression of the proteins results in the appearance of breaks along the ventral nerve cord (Fig. 5P). These findings suggest that DLimk is likely to mediate at least some of the DRok-dependent functions in the developing nervous system.

## Conclusion

In conclusion, our genetic analysis of DRok in development, using ovexpression studies in the eye, in the wing and in the CNS indicates that stringent regulation of DRok activity is required for various developmental processes, such as photoreceptor maintenance and wing vein formation. In addition, our overexpression system has revealed *zipper*, the *Drosophila *nonmuscle myosin heavy chain, as a strong genetic interactor of DRok, as seen in other reported developmental events such as dorsal closure and wing planar cell polarity, confirming that myosin II is a key downstream mediator of Rho-kinase biological effects in several morphogenetic processes. Moreover, we have shown that DRok interacts with another target protein, DLimk, to influence some other aspects of tissue morphogenesis, including CNS development.

## Methods

### *Drosophila *strains and transgenes

*Drosophila *stocks were maintained at 25°C. Generated stocks include *GMR-Drok-cat*, *UAS-Drok-cat*, *GMR-Dlimk *and *UAS-Dlimk*. The *Drok *cDNA was isolated in a yeast two-hybrid screen of a *Drosophila *embryo Matchmaker™ cDNA library in pACT2 vector (Clontech) with constitutively active RhoL63 as bait. A *GMR-Drok-cat *transgene was generated by subcloning the catalytic domain (amino acids 1–506) of DRok (DRok-cat) from the *Drok*-pBSK plasmid (*Hinc II *site) into the pGMR vector (*Stu I *site). A *UAS-Drok-cat *was then constructed by subcloning from the *Drok-cat*-pGMR plasmid (*EcoR I *site) into the pUAST vector (*EcoR I *site). The *Drosophila limk *coding sequence was subcloned into the pGMR and pUAST vectors and transformed into *Drosophila *as described previously [34]. GAL4 drivers include *engrailed*-GAL4 (*en*-GAL4), *actin5c*-GAL4, *tubulin*-GAL4, *daughterless*-GAL4 (*da*-GAL4), *prd*-GAL4, *69B*-GAL4, LE-GAL4, *elav*-GAL4, *1407*-GAL4, *Cy6*-GAL4, and *eyeless*-GAL4. Other stocks utilized include *sqh*^2 ^and *zip*^1 ^(kindly provided by Daniel Kiehart), *GMR-p35*, *hts*^1103 ^(from the Bloomington Stock Center), *dia*^*k07135 *^(Bloomington), and *Rho1*^720 ^(Bloomington).

### Microscopy and immunochemistry

For adult eye images, sections, and scanning electron micrographs (SEMs), genotypes were as follows: *Ore*^*R*^; *GMR-Drok-cat/+; GMR-Drok-cat/GMR-Drok-cat; GMR-Drok-cat*^1^*-GMR-Drok-cat*^2^/+; *zip*^1^/+, *GMR-Drok-cat*^1^*-GMR-Drok-cat*^2^/+; *sqh*^2^/+, *GMR-Drok-cat*^1^-*GMR-Drok-cat*^2^/+; *GMR-p35*/+, *GMR-Drok-cat*/+; *hts*^1103^/+, *GMR-Drok-cat*/+; *dia*^*k07135 *^*GMR-Drok-cat*/+; *GMR-Dlimk*/+; *GMR-Dlimk*/*GMR-Drok-cat*. Adult eye sections were performed according to standard protocols [35]. For adult wings, the genotypes were as follows: *engr*-GAL4/+, *UAS-Drok-cat*/+; *zip*^1^/*en*-GAL4, *UAS-Drok-cat*/+; *hts*^1103^/*en*-GAL4, *UAS-Drok-cat*/+; *rho1*^720^/*en*-GAL4, *UAS-Drok-cat*/+; *dia*^*k07135*^/*en*-GAL4, *UAS-Drok-cat*/+. Staining of dissected third instar larval imaginal eye discs was performed as described previously [35]. Staining of embryonic CNS (central nervous system) was carried out as described previously [36]. The following antibodies were used: mouse anti-Elav (1:50, Developmental Studies Hybridoma Bank – DSHB), mouse 24B10 (anti-Chaoptin) (1:50, DHSB), mouse monoclonal BP102 (1:200, DHSB). Rhodamine-phalloidin (Sigma) was used at 1:200. Immunofluorescence images were collected on a Carl Zeiss Axiovert 100 M confocal microscope.

### Mutagenesis

Mutagenesis was performed using ethyl methane sulfonate (EMS) as described previously [37]. Females carrying two copies of the *GMR-Drok-cat *transgene on each chromosome III were mated to mutagenized males and F1 male progeny was screened for suppression of the two copy-*GMR-Drok-cat*-induced rough eye phenotype. Stocks were made from each male carrying a putative suppressor mutation.

## Authors' contributions

VV performed the majority of the experimental studies and wrote much of the manuscript. GC performed the studies of Drok-Dlimk genetic interactions. JS oversaw all aspects of experimental design, interpretation of results, and editing of the manuscript.
